# The Promyelocytic Leukemia Zinc Finger Protein: Two Decades of Molecular Oncology

**DOI:** 10.3389/fonc.2012.00074

**Published:** 2012-07-17

**Authors:** Bandar Ali Suliman, Dakang Xu, Bryan Raymond George Williams

**Affiliations:** ^1^Centre for Cancer Research, Monash Institute of Medical Research, Monash UniversityMelbourne, VIC, Australia; ^2^College of Applied Medical Sciences, Taibah UniversityAl-Madinah Al-Munawarah, Saudi Arabia

**Keywords:** PLZF, cancer, leukemia, cell cycle, stem cells, apoptosis, cytokines

## Abstract

The promyelocytic leukemia zinc finger (PLZF) protein, also known as Zbtb16 or Zfp145, was first identified in a patient with acute promyelocytic leukemia, where a reciprocal chromosomal translocation *t*(11;17)(q23;q21) resulted in a fusion with the *RARA* gene encoding retinoic acid receptor alpha. The wild-type *Zbtb16* gene encodes a transcription factor that belongs to the POK (POZ and Krüppel) family of transcriptional repressors. In addition to nine Krüppel-type sequence-specific zinc fingers, which make it a member of the Krüppel-like zinc finger protein family, the PLZF protein contains an N-terminal BTB/POZ domain and RD2 domain. PLZF has been shown to be involved in major developmental and biological processes, such as spermatogenesis, hind limb formation, hematopoiesis, and immune regulation. PLZF is localized mainly in the nucleus where it exerts its transcriptional repression function, and many post-translational modifications affect this ability and also have an impact on its cytoplasmic/nuclear dissociation. PLZF achieves its transcriptional regulation by binding to many secondary molecules to form large multi-protein complexes that bind to the regulatory elements in the promoter region of the target genes. These complexes are also capable of physically interacting with its target proteins. Recently, PLZF has become implicated in carcinogenesis as a tumor suppressor gene, since it regulates the cell cycle and apoptosis in many cell types. This review will examine the major advances in our knowledge of PLZF biological activities that augment its value as a therapeutic target, particularly in cancer and immunological diseases.

## Introduction

The family of zinc finger proteins is composed of regulatory proteins that participate in many molecular and cellular pathways and is considered to be one of the most profuse regulatory protein families in eukaryotic cells, with more than 200 members. Different members of the zinc finger protein family contain multiple cysteine (Cys) and/or histidine (His) residues that require one or more zinc ions to stabilize their structures. The majority of the zinc finger proteins play important roles in DNA binding, RNA binding, RNA packaging, and protein–protein interactions. The family is divided into three sub-groups according to structure and the number of zinc fingers (Laity et al., 2001).

The zinc finger and BTB domain containing 16 (*Zbtb16*) gene was first described in humans in 1993, where its encoded protein was found to be fused in-frame with the retinoic acid receptor alpha (RARα) in a patient diagnosed with a rare form of acute promyelocytic leukemia (APL) with a reciprocal chromosomal translocation *t*(11;17). The protein was named the promyelocytic leukemia zinc finger (PLZF), because it was the first zinc finger family member exhibiting a pronounced effect on the pathology of promyelocytic leukemia (Chen et al., 1993).

In humans, the gene is localized to chromosome 11q23 among a cluster of genes, all related to the zinc finger family. The gene has three splice variants (transcripts), all encoding functional proteins, and composed of seven exons with shared homology between exons 3 and 6. The first transcript (ENST00000335953) is 2,477 bp in length and encodes a 673 aa protein. The second transcript (ENST00000392996) is 2,249 bp in length and encodes a 673 aa protein. The third transcript (ENST00000310883) is 1,728 bp in length and encodes a 550 aa protein. In mouse, the gene is located in chromosome 9, and is composed of seven exons. However, unlike its human analog, it only has one transcript (ENSMUST00000093852) that is 5,100 bp in length and encodes a 673 aa protein, which is similar in length to the human analog transcripts 2 and 3.

**Figure 1:**
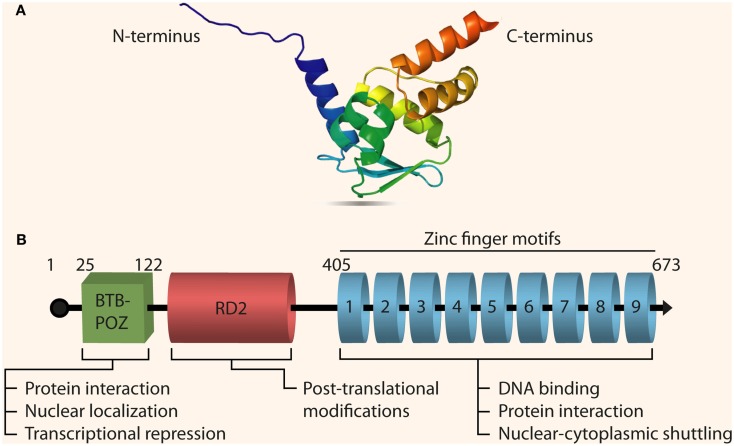
**Structure of PLZF**. **(A)** The 3D crystal structure of PLZF resolved using X-ray diffraction [PDB ID: 1CS3, Resolution (Å): 2.00; Li et al., 1999]. PLZF contains two β strands in its N-terminal sequence along with a BTB domain, and one α helix in the C-terminal with nine zinc finger motifs. **(B)** The full-length primary transcript of PLZF (Gene ID: 7704, Transcript ID: ENST00000335953) with its three functional domains: the BTB/POZ domain, the RD2 domain, and the zinc finger domain. Some of the important biological functions of each domain are also outlined. PLZF has three splice variants (transcripts), all encoding functional proteins, and composed of seven exons with shared homology between exons 3 and 6.

The N-terminus of the protein (as seen in Figure 1) contains a Drosophila-like bric à brac, tramtrack, and broad-complex domain called BTB (Zollman et al., 1994). This domain is also known as the poxvirus and zinc finger (POZ) domain and is highly conserved in mammals and plays a major role in DNA looping as well as protein dimerization and interaction to form multi-protein complexes (Bardwell and Treisman, 1994; Yoshida et al., 1999). The middle region of the protein has an RD2 domain that is less characterized and understood than the BTB/POZ domain, although mutations in this domain modulate the transcriptional activity of PLZF (Kang et al., 2003). The C-terminus of the protein contains nine Krüppel-like C_2_H_2_ zinc fingers that facilitate sequence-specific DNA binding to its target genes allowing PLZF to act as a transcriptional repressor (Li et al., 1997). The promoter region of human *PLZF* starts about 6 kb upstream of the ATG starting codon. The first TATA box in the human genomic DNA sequence for the *PLZF* gene appears at position 6,108 in the 5′ UTR. A very GC rich region is also found about 2 kb upstream of the ATG codon.

The involvement of PLZF in blocking the differentiation of promyelocytes and causing leukemia led the way for researchers to investigate its role in many biological activities regulating cellular proliferation and differentiation. Earlier reports confined the expression of *PLZF* to stem cells and early progenitor cells (Shaknovich et al., 1998), but it is now known that *PLZF* is expressed in many of the CNS cells, hematopoietic cells in the bone marrow, glandular cells in the gallbladder, islets of Langerhans in the pancreas, respiratory epithelial cells, myocytes in the heart and skeletal muscles, endometrial stroma in the uterus, glomeruli and renal tubules, glandular cells of many parts of the gastrointestinal tract, prostate, and endocrine glands (Uhlén et al., 2005).

## PLZF Animal Models

A model of *PLZF* (*ZBTB16*) gene knockout was developed in 2000 by researchers at Memorial Sloan-Kettering Cancer Center (Cornell University). They replaced the second exon of full-length PLZF with the neomycin resistance gene. *PLZF*^−/−^ mice displayed major musculoskeletal-limb defects with homeotic transformations of vertebral segments, deformed cartilage and skeleton patterning, and alterations in digit formation that is caused by an inhibition of apoptosis in hindlimb early progenitor cells in the autopod region possibly due to *Hox* gene regulation (Barna et al., 2000). Moreover, PLZF was shown to be specifically essential for spatial colinear expression of *HoxD* genes that are expressed in the hindbrain. This was achieved by the active recruitment of histone deacetylases (HDACs), possibly through the PLZF-mediated interaction with polycomb family members such as Bmi-1, to the promoters of the *Hox* gene clusters affecting only the development of the posterior regions of the limb bud (Barna et al., 2002).

Another role for PLZF also emerged after assessing *Gli3* and *PLZF* double knockout mouse embryos, where their cooperation was shown to be required for proximal cartilage condensations in the hindlimb by orchestrating the necessary dissemination of chondrocyte progenitor cells in the proximal limb bud, independent of proximal-distal patterning (Barna et al., 2005). *PLZF* expression is also important in osteoblastic differentiation, since it is an upstream regulator of core-binding factor 1 (*CBFA1*/*Runx2*). CBFA1 affects many important factors during the pluripotent differentiation of human mesenchymal stem cells (hMSCs) into the osteogenic lineage such as collagen 1A1 (COL1A1), alkaline phosphatase (ALP), and osteocalcin (OCN). Loss of PLZF expression during the early stages of pluripotent differentiation of hMSCs leads to the disruption of spinal ossification (Ikeda et al., 2005).

*PLZF*^−/−^ male mice show partial sterility, which is caused by markedly impaired spermatogenesis. Spermatozoal maturation in the seminiferous tubules was found to be distinctly decreased as a result of increased apoptosis in spermatogonial cells (Kelly and Daniel, 2006). This confirmed previous studies suggesting a dynamic role for PLZF in maintaining the undifferentiated state of spermatogonial cells, where the loss of this expression disrupts the balance between stem cell self-renewal and differentiation (Buaas et al., 2004). It was later confirmed that PLZF binds to the *c-Kit* promoter in a spatial and temporal-dependent fashion, to inhibit the maintenance and proliferation of postnatal germ cells (Filipponi et al., 2007).

*PLZF*^−/−^ mice were also found to be more prone to viral infections. Exposing neonatal wild-type and *PLZF*^−/−^ mice to the Semliki Forest virus (SFV) was lethal as measured by survival curves. However, pretreating the mice with interferon (IFN) for 6 h prior to infection protected the wild-type neonatal mice from infection, with survival times exceeding 3 weeks. On the other hand, mice lacking PLZF expression died after 6 days of infection, with viral loads in their vital organs about a thousand times higher than wild-type mice after only 48 h post-infection. This occurred despite the fact that both genotypes expressed comparable levels of IFNs after infection, showing that activation of certain anti-viral genes was impaired in the absence of PLZF. Similarly, introducing the encephalomyocarditis virus (EMCV) via intraperitoneal injections to wild-type and *PLZF*^−/−^ mice yielded equivalent results when comparing survival curves between the two genotypes (Xu et al., 2009). This demonstrates the requirement for PLZF in the IFN-mediated anti-viral innate immune response *in vivo*. The impairment of the IFN response was due to the failure to induce a subset of IFN-stimulated genes (ISGs), accounting for the fatal phenotype in *PLZF*^−/−^ mice. Moreover, IFN-induced activation of natural killer (NK) cells was also impaired in *PLZF*^−/−^ mice, which was attributed to the marked decrease in CXCL10 expression in NK-cell-rich organs in these animals (Xu et al., 2009).

## Post-Transcriptional Regulation

One of the ways the cell uses to control the expression profile of different proteins is to regulate steps in the processing of the primary RNA transcript to mature mRNA. Control of RNA processing before protein synthesis is a very effective method for gene regulation and silencing, and allows for rapid and effective silencing of gene expression. Eukaryotic cells employ many processes to achieve this type of regulation, such as m7G 5′ capping, 3′ tail polyadenylation, and intron splicing. MicroRNAs are also key regulators of post-transcriptional events via binding complementary sequences in the 5′ or 3′ UTR of their target mRNA transcripts to block translation and/or cause Ago2-dependent degradation (Hansen et al., 2011).

Promyelocytic leukemia zinc finger has been shown to regulate the expression of miR-146a *in vitro* in a PLZF-positive HEL cell line. PLZF binds directly to the promoter of *miR-146a* and inhibits its transcription, leading to an up-regulation of CXCR4 protein. CXCR4 is a chemokine that is crucially involved in the mobilization of normal hematopoietic as well as leukemic cells. It is also required for the proliferation, differentiation, and maturation of CD34^+^ megakaryocytic-progenitor cells, as demonstrated by an increase in the levels of the megakaryocytic markers CD9, CD41, and CD61(Labbaye et al., 2008).

Promyelocytic leukemia zinc finger has also been shown to target the promoter region of *miR-221/222* to inhibit their transcription in different *PLZF*-transduced melanoma cell lines. PLZF-positive melanocytes show a less malignant phenotype. Conversely, increased levels of miR-221/222, in PLZF-negative cells, correlate with increased tumorigenicity of these cells, as reflected by a decrease in G1 phase and a corresponding increase in S and G2-M phases, as well as an enhanced proliferation rate, invasiveness, motility, and anchorage-independent growth. The inhibition of these miRNAs causes an up-regulation of c-Kit, which is required for melanogenesis (Felicetti et al., 2008).

## Post-Translational Modification

### Acetylation

Acetylation is the reversible addition of an acetyl group to lysine residues in proteins, which has a major effect on their functions and interactions. Lysine residues in PLZF zinc finger 9 were shown to be acetylated by p300 both *in vitro* and *in vivo*. This acetylation is detrimental for PLZF binding to the specific DNA sequence 5′-TACTGTAC-3′, which is found in the promoters of its target genes. Mutating these lysine residues prevented PLZF from binding to this sequence and it subsequently lost its transcriptional repression, as evidenced by the failure to inhibit the growth of epithelial-like osteosarcoma cells *in vitro*. Since this process only affected the zinc fingers, which are necessary for DNA binding, neither PLZF stability nor its binding capacity to other proteins were affected (Guidez et al., 2005).

### Phosphorylation

Phosphorylation is the reversible addition of a phosphate group to serine and threonine residues of a protein, which modulates its function by prompting conformational changes in 3D structure or activation of its kinase ability. PLZF was shown to be phosphorylated by cdc2/Cyclin-B *in vitro*, which affected its DNA-binding ability to the promoter of many transcription factors such as *c-Jun* and *GATA1* (Ball et al., 1999). Cyclin-dependent kinase (CDK)-2 was also reported to phosphorylate PLZF at threonine residue 282 and serine residue 197 *in vitro* (Table 1). Unlike cdc2 phosphorylation, which directly regulates PLZF transcriptional function, CDK2-mediated phosphorylation of PLZF did not affect its nuclear localization but rather induced its ubiquitination and subsequent degradation, indirectly regulating its function. Mutation of those phosphorylation sites caused PLZF to become even more transcriptionally repressive compared to the wild-type PLZF (Costoya et al., 2008).

**Table 1:** **Post-translational modifications of PLZF**.

Modification	Effector	Site	Biological effect	Reference
Acetylation	P300	K647	Inhibits PLZF transcriptional repression through its ninth zinc finger	Guidez et al. (2005)
		K650	Modulates PLZF ability to bind specific DNA sequences	
		K653	Suppresses cellular growth	
Phosphorylation	CDK2	S197	Regulates PLZF stability and its degradation potential	Costoya et al. (2008)
		T282	Activates cyclin-A2, which drives cell cycle progression	
Sumoylation	SUMO-1	K242	Regulates PLZF transcriptional repression through its RD2 domain	Kang et al. (2003)
			Modulates PLZF DNA-binding capacity	
			Affects many PLZF biological activities	
Ubiquitination	BTBD6	BTB	Marks PLZF for proteasomal degradation and nuclear export	Sobieszczuk et al. (2010)
		domain	Antagonizes PLZF inhibition of neurogenesis	

Interferon was also reported to cause the phosphorylation of PLZF at serine residue 76 *in vitro*. This phosphorylation identified mitogen-activated protein kinase 8 (JNK) or a kinase in the same pathway to be the responsible enzyme. The study demonstrated that PLZF transcriptional activity is enhanced and caused the binding of PLZF to the promoter of many ISGs. Unlike its previously reported transcriptional repression activity, following IFN stimulation, PLZF acted as a transcriptional activator, which was a novel finding for this zinc finger protein family member (Xu et al., 2009).

### Sumoylation

Small ubiquitin-like modifier (SUMO) proteins are a family of small proteins that reversibly attach to their target proteins to regulate their functions. SUMO-1, a SUMO protein family member, binds to lysine 242 residue in the RD2 domain of PLZF *in vivo*. It abrogates the DNA-binding ability of PLZF and therefore relieves its transcriptional repression on cell cycle progression through re-activation of *cyclin-A2* (Kang et al., 2003). Another recent study has shown that metadherin (MTDH) binds to sumoylation sites in the RD2 terminal of PLZF while inside nuclear bodies. This interaction significantly hindered the ability of PLZF to bind the *c-myc* promoter and relieved its transcriptional repression. Moreover, MTDH binding to PLZF increased its complex formation with HDAC4 in favor of HDAC1, two well-known HDACs with which PLZF interacts to achieve its inhibitory effect through chromatin remodeling (Thirkettle et al., 2009). This could also explain the potential variability of the PLZF/HDAC interaction, which leads to opposite transcriptional fates depending on the post-translational modification of PLZF.

### Ubiquitination

Ubiquitin is a highly conserved small protein found only in eukaryotes. Its main function is to bind other proteins that are not needed in the cell and mark them for proteasomal degradation. Through this process (ubiquitination or ubiquitylation), ubiquitin modulates many biological processes by targeting different proteins in various pathways directing them for biological recycling. The BTB/POZ domain of PLZF was shown to bind BTBD6, a known ubiquitin ligase adaptor protein for the CUL3 ubiquitin E3 ligase complex, leading to its export from the nucleus and subsequently its degradation. The removal of PLZF from the cell was shown to be essential for differentiation of neural progenitor cells during the early stages of neurogenesis, possibly by restoring *NEUROG1* expression (Sobieszczuk et al., 2010), emphasizing its role in regulating stem cell self-renewal. Recently, an antagonistic relationship between SUMO-1 and ubiquitin for lysine residue 242 in the RD2 domain of PLZF was suggested. This was linked to abnormal physiological conditions of cells in culture, such as serum deprivation, which increase reactive oxygen species (ROS) and subsequently modulate PLZF function through either ubiquitination or sumoylation (Kang et al., 2008).

## PLZF and Cellular Biology

### Localization in the cell

After the discovery of the *PLZF* gene in 1993, the protein product was subsequently noted to be localized inside the nucleus within specialized nuclear compartments called nuclear speckles (Koken et al., 1997). Nuclear speckles or interchromatin granule clusters are specialized organelles that contain large numbers of transcription factors and are involved in pre-mRNA processing and truncation (Spector and Lamond, 2011). Many studies have shown that PLZF transcriptional repression is carried out while the protein is localized inside these nuclear compartments, and once PLZF is shuttled outside of the nuclear speckles, the repression is lost. For instance, phorbol ester 12-*O*-tetradecanoylphorbol-13-acetate (TPA) treatment of human fibrosarcoma HT1080 cells caused the metalloprotease cleavage of membrane-anchored heparin-binding EGF-like growth factor (proHB-EGF) into its soluble form HB-EGF-C. This soluble form is known to bind the zinc finger motif of PLZF and mediates its nuclear export into the cytoplasm. The decrease in PLZF nuclear level in TPA-treated HT1080 cells led to increased cyclin-A2 levels, a known PLZF-target gene, which was responsible for cell cycle progression into the S-phase (Nanba et al., 2003). Conversely, many other substances increase PLZF expression inside nuclear speckles and therefore increase its transcriptional activity, such as seen after retinoic acid and IFN treatment (Koken et al., 1999).

### Self-renewal vs. differentiation

Promyelocytic leukemia zinc finger plays a key role in both the renewal and maintenance of stem cells and early progenitor cells. Reid et al. studied early hematopoietic progenitors and showed that PLZF is expressed in high levels in undifferentiated, multi-potential hematopoietic progenitor cells. They also demonstrated how PLZF expression declined when these cells were committed to a specific hematopoietic lineage and started to differentiate (Reid et al., 1995). During early erythropoiesis, PLZF regulates both myeloid and erythroid CD34^+^ lineages as they proliferate, controlling their commitment to differentiation. PLZF binds to the promoter of *c-Kit* and inhibits its expression. c-Kit activation by its ligand, the stem cell factor (SCF), is known to be crucial for the maintenance and differentiation of hematopoietic stem cells and hematopoietic progenitor cells (Muta et al., 1995). The disruption of c-Kit by PLZF inhibits proliferation and maturation of CD34^+^ cells. Despite the negative effect of PLZF on the cell cycle of differentiated cells, high levels of PLZF are accompanied by fast growth in early hematopoietic progenitor cells, showing that not only is PLZF function dependent on cell type, but it is also required for maintaining the self-renewal capacity of the progenitor cells (Dai et al., 2002). The capacity of male germ line stem cells (spermatogonial cells) to maintain their numbers through self-renewal has also been linked to PLZF function (Buaas et al., 2004). This is thought to be achieved through the interaction of PLZF with a polycomb ring finger oncogene known as *BMI1*, which was identified to be required for hematopoietic and leukemic self-renewal ability (Park et al., 2003). A list of proteins that interact with PLZF is given in Table 2.

**Table 2:** **PLZF interacting partners**.

Protein	Association	Function	Reference
AT_2_ receptor	Protein–protein interactions	Promotes cardiac hypertrophy, and is vital for programmed cell death (apoptosis)	Senbonmatsu et al. (2003)
BCL6	Protein–protein interactions	Involved in leukemogenesis	Wong and Privalsky (1998)
CRMP-1	Complex formation	Modulates sialic acid synthesis necessary for cell–cell interactions and is involved in the organization of the cellular cytoskeleton	Weidemann et al. (2006)
CUX1	Promoter interaction	Blocks PLZF transcriptional activity *in vivo* and *in vitro*	Fréchette et al. (2010)
DRAL/FHL2	Protein–protein interactions	Augments PLZF transcriptional repression	McLoughlin et al. (2002)
EEF1A1	Protein–protein interactions	Inhibits SiHa cervical cancer cell growth by inducing apoptosis and suppressing human cyclin-A2 promoter activity	Rho et al. (2006)
ETO	Complex formation	Inhibits PLZF transcriptional repression	Melnick et al. (2000)
FAZF	Heterodimerization	Augment PLZF transcriptional repression and binds to the same target genes as PLZF	Hoatlin et al. (1999)
FLT3	Complex formation	Inhibits PLZF transcriptional repression and blocks PLZF-mediated growth suppression of leukemia cells	Takahashi et al. (2004)
GATA1	Complex formation	Plays an essential role in erythroid and megakaryocytic cell differentiation	Labbaye et al. (2002)
GATA2	Complex formation	PLZF modifies GATA2 transactivation capacity, which is implicated in the survival and growth of multi-potential progenitors	Tsuzuki and Enver (2002)
HB-EGF	Protein–protein interactions	Functions as an intracellular signal and coordinates cell cycle progression toward the S-phase	Nanba et al. (2003)
HDAC1	Complex formation	Involved in the development of both lymphoid and myeloid leukemia	David et al. (1998)
HDAC4	Complex formation	Involved in leukemogenesis	Chauchereau et al. (2004)
HDAC7	Complex formation	Represses genes responsible for maintaining myeloid lineage potential	Lemercier et al. (2002); Dequiedt et al. (2003)
HoxD	Chromatin remodeling	Required for temporal and spatial co-linearity of normal limb development	Barna et al. (2002)
mSin3	Complex formation	Required for the inhibitory complex that mediates PLZF transcriptional repression	David et al. (1998)
MTDH	Protein–protein interactions	Blocks PLZF transcriptional repression on c-Myc and increases the potential of forming complexes with HDAC4 vs. HDAC1	Thirkettle et al. (2009)
N-CoR	Complex formation	Required for the recruitment of histone deacetylase to PLZF	Huynh and Bardwell (1998)
PML/RARα	Heterodimerization with PLZF/RARα	Involved in the pathophysiology of acute promyelocytic leukemia (APL)	Ruthardt et al. (1998)
pRB	Protein–protein interactions	Regulates many processes that are deregulated in cancer, including cell cycle progression, apoptosis, and cellular differentiation	Benevolenskaya et al. (2005)
Sall4	Protein–protein interactions	Implicated in maintaining PLZF localization inside the nuclear speckles	Hobbs et al. (2012)
SMRT	Complex formation	Plays a role in the transcriptional silencing of PLZF-target genes	Hong et al. (1997)
Sp1	Protein–protein interactions	PLZF inhibits Sp1 transactivation of the epidermal growth factor receptor promoter	Vallian et al. (1998)
VDR	Protein–protein interactions	Regulation of 1,25-dihydroxyvitamin D(3) response	Ward et al. (2001)
VDUP1	Complex formation	Modulates cell cycle through cyclin-A2 promoter activity and suppresses IL-3 receptor expression	Han et al. (2003)

*Proteins that interact with PLZF to regulate its transcriptional/biological activity *in vivo* and *in vitro**.

Moreover, inactivation of PLZF in mouse models leads to the exit of spermatogonial cells from a quiescent state of regeneration and the subsequent entry into meiosis, leading to testicular degeneration as a result of increased apoptosis (Costoya et al., 2004). This dependency of stem cells and early progenitor cells on PLZF expression, which is required for embryonic tissue development, does not stop after maturation. PLZF was recently linked to the maintenance of adult stem cell populations through the modulation of Sal-like protein 4. Sall4 is another zinc finger transcription factor that is involved in embryonic stem cell pluripotency and early embryonic development by driving differentiation through c-Kit activation (Zhang et al., 2006). The POZ/BTB domain of PLZF binds Sall4 and maintains its localization inside nuclear speckles of adult stem cells. This interaction prevents Sall4 from binding to its target nucleotide sequences in heterochromatin, therefore inhibiting its transcriptional activity, which is required for differentiation (Hobbs et al., 2012).

### Cell cycle and apoptosis

The first target gene identified for PLZF was *cyclin-A2* (Yeyati et al., 1999). Cyclins are proteins that regulate the function of many small CDKs. These CDKs control the temporal coordination of the cell cycle by modulating the functions of many enzymes and transcriptional factors required for each mitotic step. cyclin-A2 in particular is very important in the transition between cell cycle phases as it regulates cdc2, which controls G_1_/S and G_2_/M checkpoints (Russo et al., 1996). Early experiments on PLZF showed that it repressed cell cycle progression in 32Dcl3 (G/GM) murine myeloid cells causing them to accumulate in S-phase. A similar result was also observed in the NB4 leukemic cell line and non-hematopoietic NIH3T3 cells. This was caused by the direct interaction of PLZF with the *cyclin-A2* promoter leading to the suppression of its expression, which was needed for both G_1_/S and G_2_/M transition. Rescuing cyclin-A2 expression in these cells successfully rescued the growth suppression effect of PLZF (Yeyati et al., 1999). Furthermore, Geminin, a known protein that initiates DNA replication in the S-phase of the cell cycle, was found to bind *Hox* regulatory DNA elements that are targeted by the BTB/POZ domain of PLZF and affect the same target genes; the *Hox* family (Luo et al., 2004).

Because PLZF expression has a pronounced impact on cell cycle progression, different studies attempted to investigate its direct role in causing apoptosis. Earlier reports showed that PLZF overexpression caused interleukin-3 (IL-3)-dependent murine hematopoietic precursor cells to accumulate in the G_0_/G_1_ phase of the cell cycle. These cells also showed levels of apoptosis in culture as assessed by TUNEL and Annexin-V staining (Shaknovich et al., 1998). In Jurkat cells, the induction of PLZF expression caused similar outcomes with a significant decrease in the number of cells in S-phase and an accumulation of cells in G_0_ phase. Additionally, more than 50% of cells stained positive for Annexin-V after only 2 days in culture. This was also accompanied by a down-regulation in the anti-apoptotic telomerase reverse transcriptase (*TERT*) gene and an up-regulation of tumor protein p53-inducible nuclear protein 1 (*TP53INP1*), inhibitor of DNA binding 1 (*ID1*), and inhibitor of DNA binding 3 (*ID3*); all of which are apoptosis-inducing genes (Bernardo et al., 2007). In a more direct observation, PLZF was shown to be responsible for up-regulating caspase-3 enzymatic activity in HeLa cervical cancer cells reflected by apoptosis-specific proteolytic cleavage of PARP in whole-cell lysates (Rho et al., 2007).

### Glucocorticoid response

Glucocorticoids (GC), such as cortisone and dexamethasone, are a type of steroid hormones that are synthesized and secreted by the adrenal cortex. They function through the GC receptors, which are found in the vast majority of mammalian cells, to regulate many essential biological functions, such as lipolysis, glycolysis, and cardiovascular and immunological processes. In a DNA microarray analysis using the T47D/A1-2 breast cancer cell line, PLZF was found to be differentially induced by dexamethasone. This induction was suggested to be, in part, the cause of breast cancer cell death after dexamethasone treatment (Wan and Nordeen, 2002). Following a discovery of the PLZF homolog in the bovine endometrium cDNA library, Fahnenstich and her colleagues were able to show that PLZF is induced in primary human endometrial stromal cells and myometrial smooth muscle cells after dexamethasone and hydrocortisone treatment *in vitro*. They also demonstrated how human endometrial and myometrial stromal cells expressed PLZF only during the mid-late secretory phase of the menstrual cycle *in vivo*, where the levels of GC and progesterone are relatively high (Fahnenstich et al., 2003).

### Chromatin remodeling

The addition of an acetyl group to lysine residues in core histones neutralizes the positive charge and dissociates neighboring nucleosomes, allowing the DNA to be lightly enfolded and readily accessible to regulatory factors such as transcription factors. This powerful transcriptional regulatory mechanism is carried out by two classes of enzymes: histone acetyltransferases (HAT) and HDAC inhibitors (HDACi). The ability to regulate the function of these enzymes to manipulate the DNA transcriptional signal in many disease backgrounds is crucial (Suliman et al., 2012).

Since HDAC1 was shown to bind the fusion protein PLZF-RARα, the attention on the PLZF repression effect was drawn toward a possible HDAC–PLZF interaction, where the modulation of HDACs may control the accessibility of transcription factors, such as PLZF, to the promoter of its target genes (Grignani et al., 1998). HDAC1, along with mSin3, binds to both the BTB/POZ as well as the RD2 domains of PLZF. This interaction was necessary for the transcriptional activity of PLZF to bind its cognate DNA sequences in HeLa Chang liver cells. Luciferase reporter constructs with PLZF-binding sites were used to assess the activity of PLZF in the presence of trichostatin A (TSA); a potent pan HDACi. A significant reduction in PLZF activity was observed when cells were treated with TSA, emphasizing the impact of HDACs on PLZF function (Hong et al., 1997).

Another study demonstrated that PLZF also interacts with HDAC4 and the transcriptional repression effect of PLZF was completely dependent on HDAC4 enzymatic activity (Chauchereau et al., 2004). Subsequently, HDAC3, 5, 6, and 7 were also shown to be part of the mSin3/SMRT/N-CoR complex, which is essential for PLZF activity (Huang et al., 2000; Kao et al., 2000; Li et al., 2000). More importantly, the HDAC interaction is not a universal phenomenon for BTB/POZ-containing proteins such as PLZF and BCL6 (Lemercier et al., 2002). In contrast, both HIC-1 and γ-FBP-B are BTB/POZ transcription factors, but they failed to interact with mSin3, N-CoR, or SMRT. They also show resistance to TSA, as their transcriptional repression is not affected by its treatment (Deltour et al., 1999). This shows that PLZF can be selectively recruited by HDACs and that they depend on its transcriptional repression capability in order to augment their biological function.

## PLZF and Immunity

Interferons are a group of cytokines produced by the host cells after infections or oncogenic transformations. They perform a fundamental function in instigating the innate immune response and coordinating the profile of the acquired immune response against a variety of immunological stimuli. IFNs function by activating signal transducer and activator of transcription (STATs) factors to induce the Janus kinase (Jak)–STAT signaling cascade. As discussed earlier, Xu et al. demonstrated that PLZF was required for the anti-viral innate immune response *in vivo*. Using two renal adenocarcinoma cell lines (RCC1 and ACHN), they showed that PLZF up-regulated many immune response genes after IFN treatment and that those genes contained putative PLZF-binding sites in their promoter regions. Many ISGs failed to respond to high levels of IFN in *PLZF*^−/−^ mice after viral infection. The expression levels of *Oas1g*, *CXCL10*, *Rsad2* (*Viperin*), and *Ifit2* were significantly lower, showing the requirement of PLZF in the induction of the anti-viral response (Xu et al., 2009).

Natural killer cells are a group of cytotoxic lymphocytes that represent a powerful tool of the innate immune system. NK cells can spontaneously lyse cells lacking self-antigen or the major histocompatibility complex (MHC) class-I. PLZF expression affects the effector function of NK cells. NK cells from *PLZF*^−/−^ mice, although constituting the same percentage of total lymphocytes as compared to the wild-types, were functionally impaired. This was reflected by their failure to spontaneously lyse Yac-1 cells (an NK cell-specific target because of their missing MHC-1). This was also true after poly I:C-mediated IFN activation. Moreover, *Granzyme B* (*GzmB*) induction after IFN activation was also affected, with a lower percentage of *PLZF*^−/−^splenic NK cells showing GzmB positive staining. This impairment of IFN activation was confirmed to be the direct result of lost PLZF expression, which was required by a specific subset of ISGs. In spite of the well-known role of PLZF as a transcriptional repressor, a novel activator role was described for the first time with regards to the IFN signaling pathway, where phosphorylated PLZF binds to the promoter regions of those ISGs and actively drives their transcription (Xu et al., 2009).

Natural killer T-lymphocyte (NKT) cells are a heterogeneous population of T cells that both express surface antigen markers designated for NK cells and recognize the Cd1d antigen that presents many glycoprotein and lipid molecules. Invariant NKT (iNKT) cells are a subgroup of NKT cells with distinctive properties. They express an invariant α chain of the T cell receptor (TCR), a specific TCR-β chain that uses the variable region 8.2, and they can respond to immunological stimuli by cytokine secretion within minutes (Matsuda et al., 2008). PLZF expression was found to be restricted to iNKT cells, with higher concentrations at early stages of thymocyte development and very low expression levels as cells differentiate toward maturation. Loss of PLZF expression in *PLZF*^−/−^ mice did not affect the number of mature iNKT cells, but did have a dramatic impact on their function. Most iNKT cells expressed low levels of CD44, CD8, NK1.1, DX5, NKG2D, CD244, CD24, and CD122. Conversely, they expressed high levels of CD69 and failed to remain in the thymus after maturation; instead accumulating in the liver, lymph nodes, and spleen. Functionally, *PLZF*-deficient iNKT cells failed to secrete both IL-4 and IFNγ after immunological challenge, a hallmark of NKT cells (Savage et al., 2008; Kreslavsky et al., 2009).

A similar scenario was observed in a subset of γδNKT cells that share phenotypic and functional properties with NKT cells and express PLZF endogenously. Vγ1 + Vδ6.3/Vδ6.4+ cells are the predominant subtype of γδNKT, which produce a repertoire of cytokines after innate immune stimuli. Loss of *PLZF* expression in those cells completely shut down their capacity to secrete these important cytokines. This functional deficiency was shown to be the result of impaired intrinsic development of thymocytes that leads to a reduction of activated CD44^Hi^CD62^Low^ cells in the bone marrow. Furthermore, TCR-mediated signaling was shown to be required for PLZF induction, which drives development of γδNKT cells (Kreslavsky et al., 2009).

## PLZF and Hematopoiesis

In 1995, a study described the relatively high expression of PLZF inside the nuclear speckles of CD34^+^ progenitor cells purified from human bone marrow. However, the expression levels of PLZF decreased in these hematopoietic cells as they became more differentiated. The study also noted the possibility of PLZF being involved in a regulatory network of transcription factors to control hematopoiesis (Reid et al., 1995).

One of the important activators of the PLZF promoter in the early stages of hematopoietic development was found to be Evi-1. Evi-1, which is usually up-regulated in many leukemias, was found to bind, and subsequently activate, the promoter of *PLZF* in HEL, KG1, and K562 cells *in vivo*. Although this interaction did not affect the expression level of PLZF, a theory of short cis-acting sequences working in a tissue-specific manner was proposed (Takahashi and Licht, 2002).

In a more recent study, researchers used a xenograft model of immunodeficient mice to study the role of PLZF in early myeloid development. They performed bone marrow transplantation of lineage^−^ (lin^−^) umbilical human cord blood-derived myeloid progenitor cells that had been transduced with *PLZF* expression/knockdown vectors. They successfully demonstrated that PLZF was able to regulate the differentiation capacity of these myeloid progenitors. PLZF achieved this through the transcriptional modulation of many key transcription factors involved in myeloid differentiation, such as *GFI-1*, C/*EBP*α, *LEF-1*, *DUSP6*, and *ID2*. This demonstrates how PLZF-controlled cellular proliferation in early hematopoiesis is essential in establishing a balance between the numbers of progenitor and fully differentiated cells (Doulatov et al., 2009).

## PLZF and Cancer

The PLZF (ZBTB16) protein was discovered in a patient with APL (which is characterized by abnormal accumulation of immature granulocytes). The cause of the disease was found to be a translocation *t*(11;17)(q23;q21) between *RAR*α and *PLZF*. The fusion proteins caused the progression of the leukemia by suppressing many retinoic acid-regulated genes, thereby locking the differentiation of granulocytes into the “promyelocyte” stage. Specifically, the patient had a chromosomal rearrangement that led to a reciprocal translocation where two fusion gene products were produced: *PLZF-RAR*α and *RAR*α*-PLZF*. *PLZF* is located in chromosome 11q23 and *RAR*α is located in chromosome 17q21. The first form of the fusion protein “*PLZF-RAR*α” is composed of full-length PLZF until the second zinc finger of the protein, where a breakpoint joins *RAR*α domains B–F. The second fusion protein *RAR*α*-PLZF* binds the A domain of *RAR*α with the remaining seven zinc fingers (3–9) of PLZF (Chen et al., 1993).

Promyelocytic leukemia zinc finger-RARα works by forming multi-protein complexes with other repressor molecules such as SMRT, Sin3, HDACs, and N-CoR to block myeloid differentiation. Chimeric mice expressing the fusion protein develop a retinoic acid-resistant leukemia that requires the addition of an HDACi to restore retinoic acid sensitivity (He et al., 1998). *In vitro*, dihydroxyvitamin D_3_ (D3) and transforming growth factor β1 (TGFβ-1) drive the differentiation of the human monocytic U937 cell line. Similarly, dimethyl sulfoxide (DMSO) can induce the differentiation of the human promyelocytic leukemia HL-60 cell line. *PLZF-RAR*α expression blocked the D3-TGF-induced monocytic and DMSO-induced granulocytic differentiation of both U937 and HL-60 cells, respectively (Ruthardt et al., 1997). These results suggest that the dominant negative effect of PLZF-RARα may involve the disruption of BTB/POZ-mediated obligate homodimerization, which can cause a loss of wild-type PLZF function. Additionally, U937 cells expressing PLZF-RARα were able to proliferate after prolonged retinoic acid treatment, which may be explained by the ability of PLZF-RARα to induce *Myc* expression and inhibit dual specificity phosphatase 6 (*DUSP6*) expression (Rice et al., 2009).

Promyelocytic leukemia zinc finger-RARα was also shown to interact with Bmi-1 and that interaction causes the fusion protein to lose its function. Both PLZF and PLZF-RARα were able to bind Bmi-1 *in vitro* to form the PRC1 polycomb complex, which can then be recruited to retinoic acid response elements (RAREs), transforming the chromatin in those regions insensitive to remodeling by HDACs. This could explain the non-responsiveness phenotype to all-trans retinoic acid treatment in PLZF-RARα-mediated APL patients (Spector and Lamond, 2011).

In a yeast two-hybrid screening, a new cervical cancer suppressor 3 (CCS3) protein was found to interact with the BTB domain of PLZF, reflecting the high expression of PLZF in normal cells and the absence of expression in cancer cells. CCS3 expression followed a similar pattern in human cervical cells. Restoring CCS3 expression in SiHa cervical cancer cells caused a dramatic change in morphology, a reduction in the numbers of viable cells in culture, and increased apoptosis. CCS3 was shown to achieve this by interacting with PLZF to repress PLZF-target genes, such as *CCNA2* (*cyclin-A2*; Rho et al., 2006).

The androgen-independent prostate cancer cell line DU145 lacks endogenous *PLZF* expression and that expression could be restored by the ectopic expression of the androgen receptor (AR). Overexpressing PLZF in these cells caused a marked down-regulation of the pre-B-cell leukemia transcription factor (PBX1) and led to decreased proliferation and survival *in vitro*. However, this was not true in LNCaP cells, which are androgen-dependent prostate cancer cells. This reflects the fact that PLZF-mediated repression is androgen-dependent and indicates how DU145 could escape androgen-based chemotherapy (Park et al., 2003).

A similar study performed on primary malignant melanoma tumors from 41 patients found that *PLZF* is expressed in the majority of tumors *in vivo*, but this expression was lost in melanoma cell lines cultured *in vitro*. The study also showed that *PBX1* expression increased as a result of low PLZF levels, which may contribute to the tumorigenesis of melanoma. Additionally, *PLZF* mRNA expression was used as a prognostic/survival index for patients (Brunner et al., 2008).

The expression of *PLZF* has been found to be frequently lost in the highly aggressive malignant mesothelioma cells as a result of recurrent focal chromosomal deletions. Restoring the expression of PLZF in these cells caused decreased viability in culture, reduced colony formation in soft agar, increased apoptosis, and an up-regulation of the cleaved Mcl-1 protein, which is a pro-apoptotic marker. Despite earlier reports connecting *PLZF* expression with *Myc* and *cyclin-A2*, no significant difference in the expression levels of these genes was noted in the study (Cheung et al., 2010).

An analysis of the upstream translated region of human *PLZF* revealed many putative binding sites for the CCAAT displacement protein (CUX1). *In vivo* interaction between CUX1 and the*PLZF* promoter was confirmed by electrophoretic mobility shift assay (EMSA) and chip analysis. CUX1 was also able to bind to the promoter region of *PLZF* in Caco-2/15 and HEK293 cells *in vitro*. A reporter assay showed that CUX1 blocked the transcriptional repression of PLZF in a dose-dependent manner. When assessing the *PLZF* gene expression profile in different colon cancer cells, an inverse relation was discovered between *PLZF* and *CUX1* gene expression (Fréchette et al., 2010).

## Discussion

**Figure 2:**
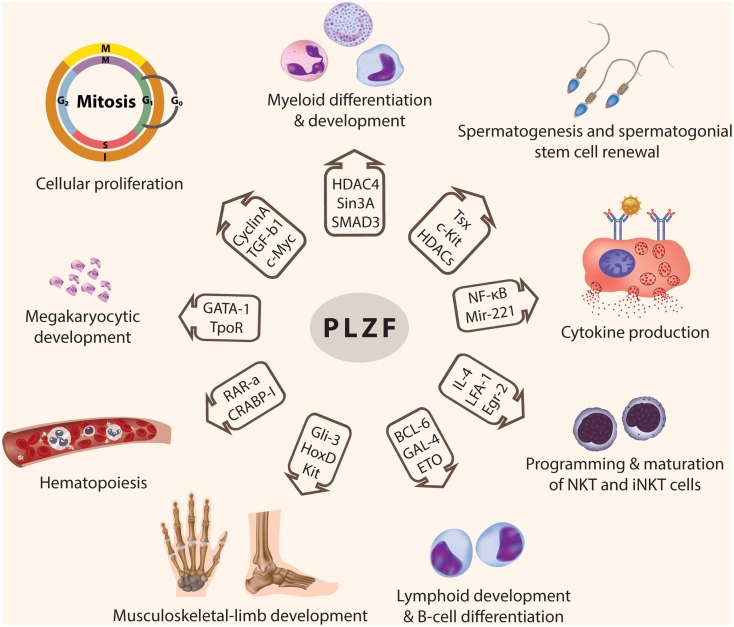
**Functions of PLZF**. PLZF is involved in the transcriptional regulation of many genes, which are responsible for modulating many developmental biological processes including: cellular proliferation and cell cycle control, myeloid and lymphoid cell development and differentiation, programming of NKT and iNKT cells, spermatogenesis and spermatogonial stem cell renewal, hematopoiesis, musculoskeletal-limb development, megakaryocytic development, and cytokine production (© 2012 Shutterstock.com).

Promyelocytic leukemia zinc finger is a transcription factor implicated in major developmental and biological processes including spermatogenesis, hind limb formation, hematopoiesis, and immune regulation (Figure 2). PLZF achieves its transcriptional regulatory activity by interacting with different partners to form large multi-protein complexes that bind to the regulatory elements in the promoter region of the target genes. Recently, PLZF has become implicated in carcinogenesis as a tumor suppressor gene, because of its ability to regulate the cell cycle and apoptosis in many cell types.

**Figure 3:**
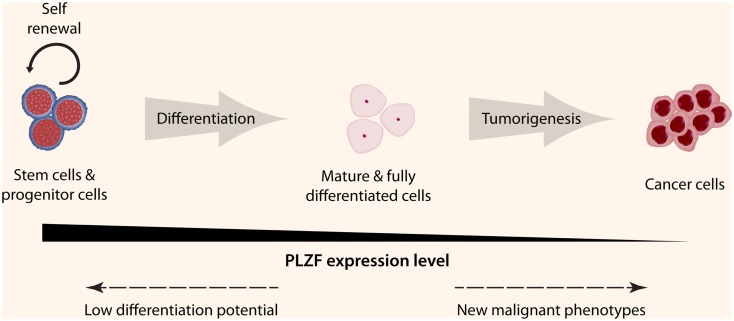
**PLZF expression levels in different cell types**. PLZF is highly expressed in stem cells and many early progenitor cells that ensure the continuation of self-renewal and low differentiation potential. This expression starts to decrease once the cell is committed to a specific lineage and PLZF is minimally expressed once the cell is fully differentiated. If a differentiated cell undergoes transformation, different mechanisms are likely involved to completely shutdown PLZF expression in order for the transformed cell to acquire new malignant phenotypes (© 2012 Shutterstock.com).

Many biological functions of PLZF in differentiated cells remain to be determined. The number of regulatory factors controlled by PLZF or by its target genes is substantial. Although expressed in many tissue types, its expression level is low compared to the levels in stem cells or progenitor cells. However, this does not mean that it has a limited function in differentiated cells, as can be seen by the large cohort of biological functions listed in Table 2. What is more intriguing is the fact that *PLZF* expression is lost in many cancer cells (Figure 3). As discussed earlier, the loss of *PLZF* expression has been correlated to many malignant phenotypes in different cancer cell types, including increased proliferation, enhanced invasiveness and motility, and resistance to apoptosis. All of these features are required for cancer cell survival, therefore *PLZF* is considered to be a tumor suppressor gene. Thus the requirement to turn off the expression of PLZF for tumorigenesis is evident, but the exact mechanism has yet to be elucidated. Possible mechanisms include epigenetic modifications or up-regulation of other transcription factors in order to silence the unfavorable PLZF expression, microRNAs to block mRNA translation, or signals to reduce the stability of the PLZF protein.

Promyelocytic leukemia zinc finger can also regulate the expression of different microRNA molecules. This ability is important, because it can aid in the understanding of how PLZF achieves the transcriptional repression of many downstream targets. This also adds an extra level of regulation to the already known promoter-dependent transcriptional regulatory activities of PLZF. A single microRNA molecule not only can target a single pre-mature mRNA, but also can regulate a plethora of mRNA molecules, leading to an amplified transcriptional regulatory signal that could affect the basic properties of the cell, including proliferation rate, apoptosis resistance, migration, and invasiveness.

Understanding the biological activities of PLZF and its DNA/protein/miR interaction network would augment our understanding of its complex involvement in many pathways and may delineate the potential of fine tuning its expression profile in cancer cells. This would add to its value as a therapeutic target to modulate malignant cellular characteristics in oncological disease backgrounds.

## Conflict of Interest Statement

The authors declare that the research was conducted in the absence of any commercial or financial relationships that could be construed as a potential conflict of interest.
